# Supplementary material for: Inhibition of p300/CBP-Associated Factor Attenuates Renal Tubulointerstitial Fibrosis through Modulation of NF-kB and Nrf2
Source: Int J Mol Sci. 2019 Mar 28;20(7):1554. doi: 10.3390/ijms20071554 (PMC6479343; doi:10.3390/ijms20071554)
Supplement: Supplementary file 1 [file ijms-20-01554-s001.pdf]

Supplementary Material: Inhibition of p300/CBP-Associated Factor Attenuates Renal Tubulointerstitial Fibrosis through Modulation of NF-kB and Nrf2

Sungjin Chung[1], Soojeong Kim[2], Mina Son[1], Minyoung Kim[1], Eun Sil Koh[1], Seok Joon Shin[1], Cheol Whee Park[1] and Ho-Shik Kim[2,*]

[1] Department of Internal Medicine, College of Medicine, The Catholic University of Korea, 222, Banpo-daero, Seocho-gu, Seoul 06591, Republic of Korea; chungs@catholic.ac.kr (S.C.); sma0917@naver.com (M.S.); gomy33@naver.com (M.K.); fiji79@catholic.ac.kr (E.S.K.); imkidney@catholic.ac.kr (S.J.S.); cheolwhee@hanmail.net (C.W.P.)

[2] Department of Biochemistry, College of Medicine, The Catholic University of Korea, 222, Banpo-daero, Seocho-gu, Seoul 06591, Republic of Korea; soojeong107@hanmail.net (S.K.); hoshik@catholic.ac.kr (H.-S.K.)

* Correspondence: hoshik@catholic.ac.kr; Tel.: +82-2-2258-7294

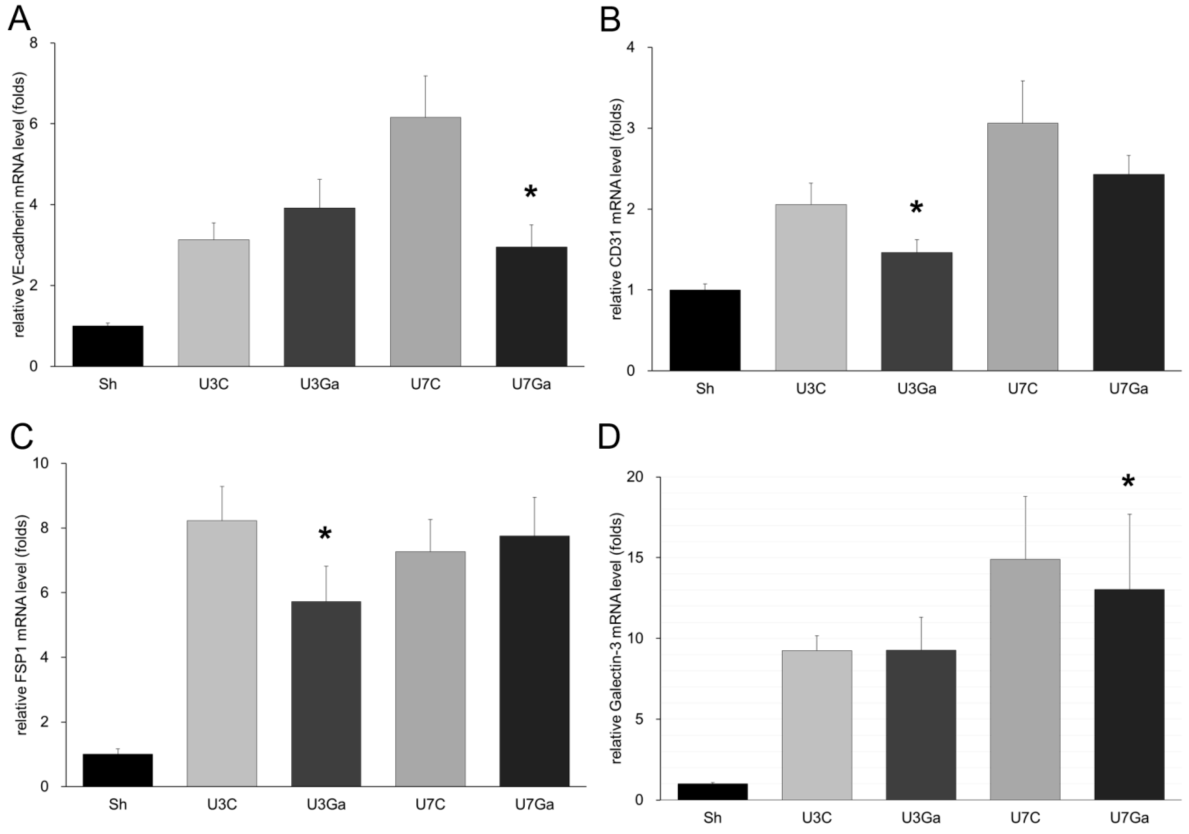

Figure S1. Renal mRNA levels of vascular endothelial or tubular epithelial makers with or without garcinol treatment after UUO. (**A**) Renal VE-cadherin mRNA level was upregulated by UUO. With garcinol treatment, its level was decreased at day 7 post-UUO. *P = 0.005 vs. U7C. (**B**) Renal mRNA level of CD31 was significantly decreased by garcinol at day 3 post-UUO. *P = 0.010 vs. U3C. (**C**) Renal mRNA level of FSP-1 was significantly elevated in the obstructed kidneys but significantly decreased by garcinol at day 3 post-UUO. *P = 0.006 vs. U3C. (**D**) Renal mRNA of Galectin-3, significantly increased in response to injury induced by obstruction, was decreased by garcinol at day 7 post-UUO. *P = 0.006 vs. U7C.

Table S1. Sequences of primers used for qRT-PCR.

Gene	Forward	Reverse
TGF-β1	GAC CGC AAC AAC GCC ATC TA	GGC GTA TCA GTG GGG GTC AG
α-SMA	GGA GAA GCC CAG CCA GTC GC	AGC CGG CCT TAC AGA GCC CA
Vimentin	CGG AAA GTG GAA TCC TTG CA	CAC ATC GAT CTG GAC ATG CTG T
Fibronectin	GAA GTC GCA AGG AAA CAA GC	GTT GTA GGT GAA CGG GAG GA
MMP-2	GCC TCA TAC ACA GCG TCA ATC TT	CGG TTT ATT TGG CGG ACA GT
MMP-9	CCT GGA ACT CAC ACG ACA TCT TC	TGG AAA CTC ACA CGC CAG AA
E-cadherin	ATC CTC GCC CTG CTG CTT	ACC ACC GTT CTC CTC CGT A
IL-6	TGT ATG AAC AAC GAT GAT GCA	GGT ACT CCA GAA GAC CAG AGG AAA T
TNF-α	CCC AGA CCC TCA CAC TCA GAT C	CCT CCA CTT GGT GGT TTG CT
VE-cadherin	GTT CAA GTT TGC CCT GAA GAA	GTG ATG TTG GCG GTG TTG T
CD31	GAC TCA CGC TGG TGC TCT ATG C	TCA GTT GCT GCC CAT TCA TCA
FSP-1	GGA GCT GCC TAG CTT CCT G	GCT GTC CAA GTT GCT CAT CA
Galectin-3	TTG AAG CTG ACC ACT TCA AGG TT	AGG TTC TTC ATC CGA TGG TTG T
